# Multiplex, DNase-free one-step reverse transcription PCR for *Plasmodium* 18S rRNA and spliced gametocyte-specific mRNAs

**DOI:** 10.1186/s12936-017-1863-3

**Published:** 2017-05-19

**Authors:** Amelia E. Hanron, Zachary P. Billman, Annette M. Seilie, Tayla M. Olsen, Matthew Fishbaugher, Ming Chang, Thomas Rueckle, Nicole Andenmatten, Bryan Greenhouse, Emmanuel Arinaitwe, John Rek, Smita Das, Gonzalo J. Domingo, Kelly Shipman, Stefan H. Kappe, James G. Kublin, Sean C. Murphy

**Affiliations:** 1 Department of Laboratory Medicine, University of Washington, 750 Republican St., E630, Seattle, WA 98109 USA; 2 Department of Microbiology, University of Washington, 1959 NE Pacific St., Seattle, WA 98195 USA; 3 Center for Emerging and Re-emerging Infectious Diseases, University of Washington, 750 Republican St., Seattle, WA 98109 USA; 4 Center for Infectious Disease Research, 307 Westlake Ave. N, #500, Seattle, WA 98109 USA; 5 Medicines for Malaria Venture, PO Box 1826, 20, Route de Pré-Bois, 1215 Geneva, Switzerland; 6 Division of HIV, Infectious Diseases and Global Medicine, Department of Medicine, University of California San Francisco, San Francisco, USA; 7 Infectious Diseases Research Collaboration, 2C Nakasero Hill Road, PO Box 7475, Kampala, Uganda; 8 London School of Hygiene & Tropical Medicine, Keppel Street, London, WC1E 7HT UK; 9 PATH, 2201 Westlake Ave #200, Seattle, WA 98121 USA; 10 Seattle Malaria Clinical Trials Center, Fred Hutch Cancer Research Center, 1100 Fairview Ave. N., #E3-300, Seattle, WA 98109 USA

**Keywords:** Gametocyte, mRNA, Spliced, Antisense, *Plasmodium*, DNase

## Abstract

**Background:**

*Plasmodium* gametocytes are sexual stages transmitted to female *Anopheles* mosquitoes. While *Plasmodium* parasites can be differentiated microscopically on Giemsa-stained blood smears, molecular methods are increasingly used because of their increased sensitivity. Molecular detection of gametocytes requires methods that discriminate between asexual and sexual stage parasites. Commonly tested gametocyte-specific mRNAs are pfs25 and pfs230 detected by reverse transcription polymerase chain reaction (RT-PCR). However, detection of these unspliced mRNA targets requires preceding DNase treatment of nucleic acids to eliminate co-purified genomic DNA. If gametocyte-specific, spliced mRNAs could be identified, DNase treatment could be eliminated and one-step multiplexed molecular methods utilized.

**Results:**

Expression data was used to identify highly-expressed mRNAs in mature gametocytes that were also low in antisense RNA expression in non-gametocyte stages. After testing numerous candidate mRNAs, the spliced female Pf3D7_0630000 mRNA was selected as a *Plasmodium falciparum* gametocyte-specific biomarker compatible with *Plasmodium* 18S rRNA RT-PCR. This mRNA was only detected in samples containing mature gametocytes and was absent in those containing only asexual stage parasites or uninfected human blood. PF3D7_0630000 RT-PCR detected gametocytes across a wide range of parasite densities in both spiked and clinical samples and agreed with pfs25 RT-PCR, the gold standard for RT-PCR-based gametocyte detection. PF3D7_0630000 multiplexed with *Plasmodium* 18S rRNA RT-PCR was more sensitive than other spliced mRNA targets for one-step RT-PCR gametocyte detection.

**Conclusions:**

Because the spliced target does not require DNase treatment, the PF3D7_0630000 assay can be multiplexed with *Plasmodium* 18S rRNA for direct one-step detection of gametocytes from whole human blood.

**Electronic supplementary material:**

The online version of this article (doi:10.1186/s12936-017-1863-3) contains supplementary material, which is available to authorized users.

## Background

*Plasmodium* gametocytes are the male and female sexual stages of the parasite responsible for transmission from an infected host into the female *Anopheles* mosquito vector. While gametocytes do not directly cause clinical disease in the mammalian host, their presence denotes potential continued transmission. Mature gametocytes circulate in peripheral blood for 3 weeks or longer [1–4]. The frequency and density of gametocyte carriage is correlated with the likelihood that mosquitoes will become infected after taking a blood meal [5–7]. Persons with sub-microscopic *Plasmodium falciparum* gametocyte densities can contribute to transmission [2, 7–9] with transmission possible at densities as low as 250–300 gametocytes/mL of blood [9]. Thus, gametocyte detection strategies should ideally be able to achieve this level of analytical sensitivity.

Gametocytes can be identified by light microscopy of Giemsa-stained thick and thin blood smears but, like all microscopic methods for *Plasmodium* parasites, only to a density of ~5000–20,000 parasites/mL (5–20/μL) by thick blood smear [10]. Molecular methods are more sensitive and include qualitative or quantitative RT-PCR [3, 11–14] and NASBA [9, 15, 16]. mRNA-based methods are used to differentiate gametocytes from asexual stages by detecting stage-specific mRNAs. The most common gametocyte mRNA targets are pfs25 [17, 18] and pfs230 [18–20] for *P. falciparum* and pvs25 for *P. vivax* [17]. These well-studied targets are all derived from unspliced mRNAs, so a DNase treatment step is required to destroy genomic DNA prior to RT. When manual DNase treatment is performed, there is partial loss of sample material and increased risk for sample cross-contamination due to added handling steps. DNase treatment also makes the process more time consuming. For detection of asexual stage parasites, some laboratories already perform one-step multiplex *Plasmodium* 18S rRNA RT-PCR directly from extracted whole blood without DNase treatment [21, 22]. In these assays, DNase treatment is not required because *Plasmodium* 18S rRNAs are more than three orders of magnitude more abundant than the coding 18S rDNA genes [21, 22]. *Plasmodium* 18S rRNAs are developmentally regulated between sexual and asexual stages [23–25] but gametocytes express both S- (sexual) and A- (asexual)-type 18S rRNAs [18]. Because of this expression, 18S rRNAs alone cannot be used to differentiate gametocytes from asexual stage parasites. Spliced mRNAs that were highly expressed in gametocytes and showed nearly absent antisense RNA expression in the asexual stage were hypothesized to be ideal targets for multiplexing with the *Plasmodium* 18S rRNA assay for one-step RT-PCR *Plasmodium* detection. Although three spliced gametocyte-expressed mRNAs have been reported as RT-PCR targets [26, 27], it was unknown whether these targets were suitable for multiplexing with the 18S rRNA assay.

Here, bioinformatic methods were used to search for spliced, gametocyte-specific mRNA targets compatible with one-step *Plasmodium* 18S rRNA RT-PCR methods. After testing candidate targets and identifying several gametocyte-specific spliced mRNAs, the PF3D7_0630000 mRNA was selected as a *P. falciparum*-specific spliced mRNA for RT-PCR without DNase treatment. This novel mRNA target was then tested against pfs25 and a known spliced mRNA using clinical samples.

## Methods

### Bioinformatics

mRNA expression fold-change from Lopez-Barragan et al. [28] was evaluated. Fold-change data was summed for asexual or gametocyte stages, sorted and filtered to remove mRNAs with total asexual expression ≥0 or total gametocyte expression ≤0 (all relative to ring stage expression). Single exon genes were eliminated as were those lacking data in http://www.plasmodb.org and those representing redundant mRNA isoforms. Fold change data in [28] were based on RNASeq reads per kilobase of exon per million (RPKM). Since RPKM can originate from sense or antisense transcripts, the strand-specific fragments per kilobase of exon per million (FPKM) [28] were also inspected, and genes with schizont antisense FPKM equal to 50–100% of maximum were eliminated as were those with minimum asexual antisense FPKM within twofold of the maximum gametocyte antisense FPKM values. Remaining genes were sorted on stage V gametocyte expression, and the most highly expressed genes were evaluated for suitable intron-spanning splice RT-PCR designs. RPKM and FPKM data deposited in plasmodb.org from [28] for selected genes is in Additional file 1.

### Primer/probes

Primers and probes were designed to target exons using the web-based PrimerQuest tool (http://www.idtdna.com/Primerquest/). Primers were commercially synthesized (IDT DNA, Coralville, IA, USA); primer set names (S1, S2, S3, etc.) were arbitrarily assigned by the IDT PrimerQuest software.

### *Plasmodium falciparum* cultures

Asexual stage *Plasmodium* parasites were cultured as described [21]. Gametocytes were cultured as described [29]. Purity of cultures was confirmed by microscopy. Some asexual stage cultures were further confirmed gametocyte-free by pfs25 RT-PCR.

### Human clinical samples

Leftover clinical whole blood specimens (50 μL) were preserved in 2 mL of NucliSens Lysis Buffer (bioMérieux, Marcy-l’Étoile, France). These samples were used under a protocol approved by the University of Washington Institutional Review Board (IRB) (protocol 47026, S. Murphy). Samples were included from a clinical trial of a novel chemical entity DSM265 approved under Fred Hutchinson Cancer Research Center IRB (protocol 8408, J. Kublin). Samples from a field study in Uganda were collected from consenting study participants or with the assent of children and consenting legal care givers within the context of studies approved by the relevant IRBs. Specimens from Nagongera, Tororo District, Uganda were collected under a study approved by the University of California San Francisco IRB 11-05995 and Uganda IRB 2011-0167. In Uganda, EDTA whole blood samples were collected from a previously described surveillance cohort [30]; all participants provided informed consent. Samples from Uganda were collected from May to October 2015.

### Nucleic acid extraction

Nucleic acids were extracted on a bioMérieux EasyMAG [21] or an Abbott m2000sp as described [22]. Briefly, on the bioMérieux instrument, 1 mL of the 2.05 mL lysed sample (50 µL whole blood plus 2 mL NucliSENS lysis buffer) was extracted for total nucleic acids and eluted into 40 µL of elution buffer. On the Abbott m2000sp instrument, 1 mL of the 2.05 mL lysed sample was extracted preferentially for RNA and eluted into 53 μL of elution buffer.

### RT-PCR

In initial studies of candidate primers, singleplex RT-PCR was performed using the AgPath mastermix kit as described by the manufacturer (Thermo Fisher Scientific, Waltham, MA) in the presence of LCGreen DNA-binding dye as described by the manufacturer (BioFire, Salt Lake City, UT). Some such RT-PCR products were evaluated by 1.5% agarose gel electrophoresis. For multiplex studies of spliced mRNA targets with 18S rRNA, pan-*Plasmodium* 18S rRNA RT-PCR was performed as described [31] with the addition of the candidate spliced gametocyte primers (200 nM) and probe (100 nM) on an Abbott m2000rt or Bio-Rad C1000 (Bio-Rad, Hercules, CA). Briefly, 15 µL of eluate was combined with 35 µL of mastermix as described in the SensiFAST LO-ROX one step RT-PCR kit product manual (Bioline, Tauton, MA). Cycling conditions were reverse transcription 10 min at 48 °C, 95 °C for 2 min followed by 45 cycles of 95 °C for 5 s followed by 50 °C for 35 s. For pfs25/18S rRNA multiplex RT-PCR, total nucleic acids were DNase treated (TURBO DNA-free Kit, Ambion/Life) and pfs25 and 18S rRNA RT-PCR performed using published cycling conditions and concentrations of *Plasmodium* 18S rRNA primers/probe [31] supplemented with pfs25 primers (400 nM) and probe (200 nM) [17]. Reagents for a gametocyte-specific mRNA from *P. falciparum* meiotic recombination protein DMC1-like protein gene (PF3D7_0816800) were as previously reported [26]: forward 5′-ATATCGGCAGCGAAAATGTGT-3′; reverse 5′-GACAATTCCCCTCTTCCACTGA-3′; probe 5′-(6-FAM)-TGCCCTTCTCGTAGTTGATTCGATTATT(BHQ1)-3′. Reagents for the early/mid- (PF3D7_1477700/PF14_0748) and mid/late-gametocyte expressed mRNAs (PF3D7_1438800/PF14_0367) were as previously reported [27]: PF3D7_1477700: forward 5′-CTTATGTGCTGAATTTTGTGTTATGGT-3′, reverse 5′-TTGGCCACACTGCTCTAGGA-3′, probe: 5′(VIC)-CACATAATGAATTCAAGGGTAG(MGBNFQ)-3′; PF3D7_1438800: forward 5′- GTTACATTTCGACCCAGCATAAATT-3′, reverse 5′-TCCCTGTGTTTTTGCTCATCTTC-3′, probe 5′-(VIC)-CAGTGCATATTGTTGCCTGT(MGBNFQ)-3′. All probes were 6-FAM-labelled and sourced from IDT (Coralville, IA) with the exceptions of the CAL Fluor orange 560-labeled pan-*Plasmodium* 18S rRNA hydrolysis probe (LCG Biosearch Technologies, Novato, CA) and the VIC-labeled PF3D7_1477700 and PF3D7_1438800 probes (Invitrogen). Where indicated, some reactions were tested at other annealing temperatures to investigate specificity. Quantification of parasites was achieved using an absolute 18S rRNA standard curve as described [22, 31], which allowed estimation of gametocyte-specific assay limits of detection (LOD). For gametocyte markers evaluated using whole parasite standard curves, C_T_ values up to 40.0 cycles were converted into quantitative values.

### Statistics

To evaluate unlysed sample stability, an unpaired t test was used to compare mean C_T_ values for 18S rRNA and gametocyte-specific markers and the difference in these C_T_ values. For sensitivity analyses, exact confidence intervals were calculated [32]. Statistical significance for both was P < 0.05.

## Results

### Target selection

One-thousand one-hundred twenty-nine genes evaluated previously by RNAseq [28] were filtered to remove those with increased asexual expression (sum of asexual fold change ≥0) and decreased gametocyte expression (sum of stage II and V gametocyte fold change ≤0). This resulted in 372 genes with gametocyte stage II and/or V expression and an absence of asexual-stage expression. The most well-known gametocyte target *pfs25* (*PF3D7_1031000*) was one of the most highly expressed gametocyte-specific genes in this set (Additional file 1). Six genes were eliminated because they lacked data in plasmodb.org and all single exon genes were also eliminated. Two-hundred genes contained two or more exons. Since antisense transcripts are produced for many *Plasmodium* genes [28] and since such transcripts can lead to false positive results in primer-specific RT-PCRs [33–36], antisense strand-specific FPKM data [28] were evaluated next. Genes with low or absent antisense transcript expression in non-gametocyte stages were retained (48 genes), sorted on stage V gametocyte expression and evaluated for intron-spanning primer/probe designs compatible with the *Plasmodium* 18S rRNA RT-PCR cycling temperatures. RT-PCR designs for 14 genes were ultimately selected using this strategy as were designs for four additional genes overexpressed in stage II/V gametocytes but not identified by published fold-change data in [28]; published RT-PCRs for pfs25 and three reported multi-exon gametocyte-specific genes [26, 27] were also included (Additional file 1). Based on recently published data [37], two genes were male-specific (PF3D7_1477700, PF3D7_1438800), one showed mixed expression (PF3D7_1020100) and the rest were female-specific (Additional file 1).

### RT-PCR with LCGreen intercalating dye for candidate targets

**Fig. 1:**
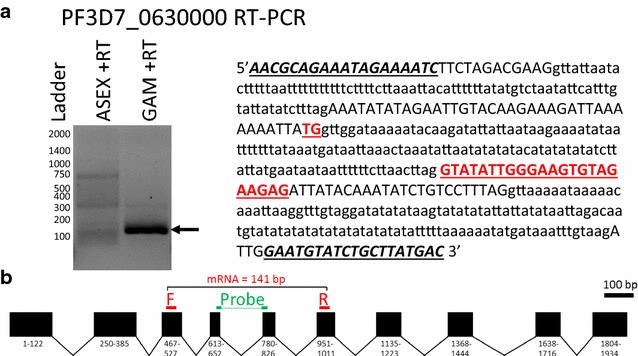
Specific amplicon produced by RT-PCR targeting gametocyte-specific spliced PF3D7_0630000 mRNA. **a** RT-PCR was performed with (+RT) and without (−RT) reverse transcriptase on nucleic acids extracted from mature gametocytes (GAM) or from asexual-stage infected red blood cells (ASEX) and products were separated by 1% agarose gel electrophoresis. **a** 100 bp ladder is shown at *left*. **b** PF3D7_0630000 mRNA

LCGreen RT-PCR was performed on 27 target regions of the 18 novel genes of interest using total nucleic acids obtained from either cultured mature *P. falciparum* gametocytes or from cultured synchronized ring-stage asexual parasites (Additional file 2). Although these RT-PCR assays do not provide absolute quantification, the difference (Δ) between C_T_ values for the 18S rRNA and the mRNA of interest provide an estimate of spliced mRNA abundance. In cultured samples enriched for mature gametocytes, gametocyte-specific spliced mRNAs were detected 6.9–25.2 cycles later than *Plasmodium* 18S rRNA (ΔC_T Gam mRNA–18SrRNA_). In cultured samples containing only asexual-stage parasites, the ΔC_T iRBC mRNA–18SrRNA_ was typically larger than the aforementioned ΔC_T Gam mRNA–18SrRNA_ for each candidate mRNA, as expected for gametocyte-expressed mRNAs (Additional file 2). mRNAs with the smallest ΔC_T Gam mRNA–18SrRNA_ and the largest ΔC_T iRBC mRNA–18SrRNA_ were considered to represent the best balance between high and specific gametocyte expression. Gel electrophoresis was used to evaluate some RT-PCR products. For example, for PF3D7_0630000, a band of the expected size (141 bp) was only generated in the presence of gametocytes by RT-PCR (Fig. 1a). The probe for this target was designed to span an intron (Fig. 1b).

### Probe-based RT-PCR

**Fig. 2:**
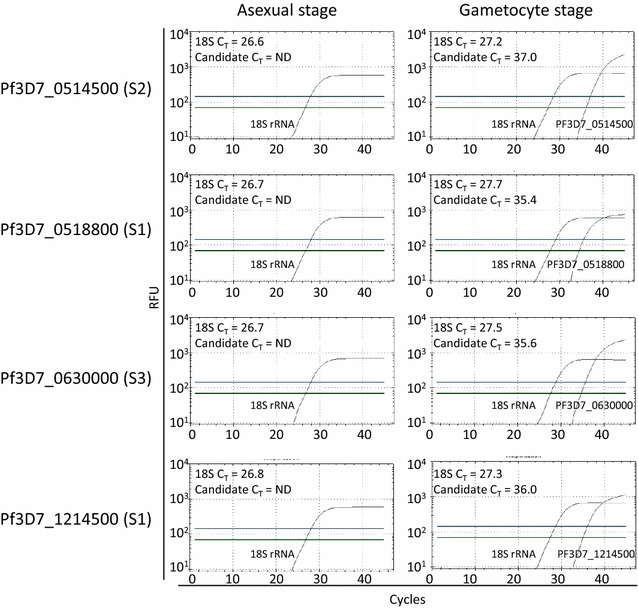
Specific detection of spliced gametocyte-specific mRNAs by hydrolysis probe RT-PCR. RT-PCR was performed using hydrolysis probes for the four spliced gametocyte-specific targets listed on nucleic acids extracted from asexual-stage infected red blood cells (*left panels*) or from mature gametocytes (*right panels*). Reactions were multiplexed with a primer/probe set for the *Plasmodium* 18S rRNA. *Horizontal lines* denote the RT-PCR thresholds used to calculate C_T_ values (*lower green line* 18S rRNA; *upper blue line* candidate mRNA). Y-axis, RFU (raw fluorescence units) from Biorad C1000/CFX instrument. *ND* not detected

Four primer sets that specifically detected gametocytes by LCGreen, had RT-PCR efficiencies of 90–110% and had probe melting temperatures compatible with the 18S rRNA RT-PCR [PF3D7_0514500 (S1), PF3D7_0518800 (S1), PF3D7_0630000 (S3), PF3D7_1214500 (S1)] were further evaluated using hydrolysis probes (Table 1). These RT-PCRs detected gametocyte mRNA in gametocyte-containing samples (Fig. 2). However, PF3D7_1214500 (S1) reacted with some parasite-negative human blood samples and PF3D7_1214500 (S1) and PF3D7_0518800 (S1) occasionally gave false positives for blood from microscopically-pure cultured asexual-stage parasites, so further study of these targets was discontinued. Positive C_T_s for PF3D7_0630000 (S3) RT-PCR were ~2–4 cycles earlier than for PF3D7_0514500 (S2) so PF3D7_0630000 was prioritized for development.

**Table 1:** Candidate primer/probe combinations evaluated for gametocyte-specific targets

Gene ID	# Exons	Exons targeted	Forward primer	Probe (±strand)	Reverse primer
PF3D7_1214500 (S1)	6	2–4	TGGGATGATGAATATGAAG	TGGAACTGGTTCGCTATATTGT (−)	AAAGGTCTATTAGTTGAATTG
PF3D7_0630000 (S3)	10	3–6	AACGCAGAAATAGAAAATC	TGGTATATTGGGAAGTGTAGAAGAG (+)	GTCATAAGCAGATACATTC
PF3D7_0514500 (S2)	6	2–3	GATAAGACAAAACGGAAC	CACAGGAGTAGTGACCATATCAG (+)	CTATAAGGAAAAGATAACAAAG
PF3D7_0518800 (S1)	7	1–2	GCCGTTACTGATTCCTTAC	TGTCTGAGTTACGACAAGAAATTAG (+)	CGAGAATACCCATTTGTC
PF3D7_1031000/pfs25	1	1	GAAATCCCGTTTCATACGCTTG	TGTAAGAATGTAACTTGTGGTAACGGT (+)	AGTTTTAACAGGATTGCTTGTATCTAA

### PF3D7_0630000-specific qRT-PCR

**Fig. 3:**
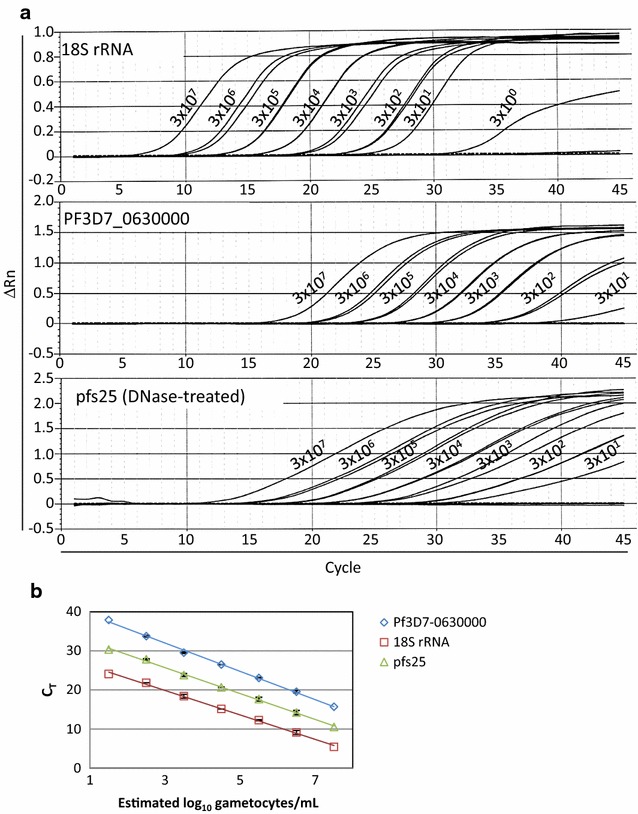
Dilution series of gametocytes tested by RT-PCR for 18S rRNA, Pf3D7_0630000 and pfs25. **a** RT-PCR was performed with hydrolysis probes on nucleic acids from enriched gametocyte cultures diluted into whole human blood across the range of parasite densities indicated in the figure (3 × 10^7^ gametocyte/mL blood to 3 gametocytes/mL blood). Samples for 18S rRNA and PF3D7_0630000 were not treated with DNase; samples for pfs25 RT-PCR were DNase treated as required for an unspliced target. DNase-treated 18S rRNA C_T_ values were comparable to non-DNase-treated C_T_s indicating that little to no 18S rRNA was lost in the DNase treatment (data not shown). Duplicates shown with Y-axis = ΔRn (change in fluorescence) from the Abbott m2000rt instrument. Labels indicate estimated parasite densities in parasites/mL (see Table 2 for log_10_ values used in panel **b**. **b** Linear regression was performed using nominal log_10_ transformed parasite densities and mean C_T_s (*error bars* 95% confidence intervals for available duplicate samples) to generate standard curves for the enriched gametocyte material

PF3D7_0630000 (aliases MAL6P1.128 and PFF1455c) encodes a CPW-WPC family protein. CPW-WPC protein-coding mRNAs are expressed and post-transcriptionally regulated in gametocytes and later translated into surface proteins in ookinetes [38]. The PF3D7_0630000 (S3) reagents target exons 3–6 of the mature mRNA with the probe and reverse primer spanning introns 4 and 5, respectively (Fig. 1b). The spliced mRNA amplicon was 141 bp. Nucleic acids from a dilution series of mature gametocytes (Day 14 of culture) were tested using RT-PCRs for *Plasmodium* 18S rRNA, PF3D7_0630000 and pfs25 to generate a standard curve and evaluate assay efficiency and linearity (Fig. 3a; Table 2). *Plasmodium* 18S rRNA qRT-PCR was able to quantitatively detect as few as 30 parasites/mL of whole blood, consistent with the assay limit of detection of 20 parasites/mL; the C_T_ curve generated for the most dilute sample was below the assay limit of quantification. Probe-based qRT-PCR for pfs25 and PF3D7_0630000 detected gametocytes from 3 × 10^7^ to 3 × 10^2^ parasites/mL, and pfs25 additionally detected parasites at the 3 × 10^1^ parasites/mL dilution. PF3D7_0630000 mRNA was never detected in samples containing asexual-stage parasites (even at high parasite densities) confirming that this spliced target is absent from asexual stages. All reactions demonstrated high cycling efficiencies (Fig. 3b: 18S rRNA slope −3.14, efficiency 108%, y-intercept: 29.4 cycles; pfs25 slope −3.32, efficiency 100%, y-intercept 35.71 cycles; PF3D7_0630000 slope −3.59, efficiency 90%, y-intercept 42.67 cycles). Consistent with RNAseq data [28], PF3D7_0630000 was less abundant than pfs25, and both mRNAs were much less abundant than the highly expressed *Plasmodium* 18S rRNA.

**Table 2:** Cycle thresholds for *Plasmodium* 18S rRNA, PF3D7_0630000 and pfs25 in gametocyte dilution series

Gametocytes/mL	*Plasmodium* 18S rRNA	PF3D7_0630000	pfs25
3 × 10^7^	5.40	15.70	10.53
3 × 10^6^	9.36	19.70	14.48
	8.85	19.38	13.94
3 × 10^5^	12.16	23.13	17.91
	12.34	22.98	17.42
3 × 10^4^	15.12	26.47	20.70
	15.11	26.50	20.65
3 × 10^3^	18.14	29.43	23.60
	18.63	29.60	23.99
3 × 10^2^	21.85	33.86	27.74
	21.82	33.65	27.92
3 × 10^1^	24.06	37.89	30.35
	ND	ND	ND
3 × 10^0^	ND	ND	ND
	ND	ND	ND

*Plasmodium* 18S rRNA RT-PCR LOD = 20 parasites/mL

*ND,* not detected; All in duplicate except 3 × 10^7^ gametocyte/mL sample

### PF3D7_0630000 mRNA stability

**Fig. 4:**
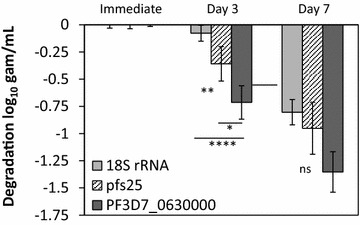
Stability of the PF3D7_0630000 mRNA compared to that of pfs25 and 18S rRNA. Samples were tested across a range of parasite densities (3 × 10^2^–3 × 10^5^ parasites/mL) and the mean change in estimated gametocyte concentration was assessed after variable storage times. *Error bars* show 95% CI; n = 8 samples for Days 0 and 3 and n = 3 samples for day 7

To assess spliced mRNA target stability, intact gametocytes in whole blood were either preserved in lysis buffer immediately or held at room temperature for 3–7 days before processing into lysis buffer. For all samples (nominal densities 3 × 10^2^–3 × 10^5^ gametocytes/mL), mRNA (pfs25 and PF3D7_0630000) degradation was observed at 3 and 7 days of storage, whereas statistically significant 18S rRNA degradation was not observed until 7 days of storage (Fig. 4). PF3D7_0630000 degraded more rapidly than pfs25 at 3 days but this difference was not statistically different at 7 days. These data suggest, while samples for 18S rRNA testing can be stored for several days prior to sample processing, samples for gametocyte-specific pfs25 or PF3D7_0630000 mRNA testing should be processed immediately following collection.

### Kinetics of PF3D7_0630000 mRNA expression

**Fig. 5:**
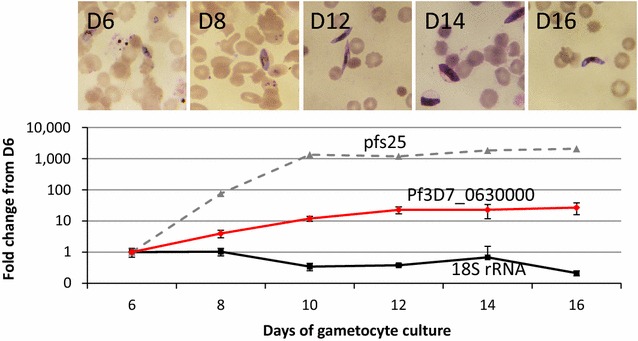
Time course of mRNA expression in cultured gametocytes. Gametocytes were cultured and equal volume and equal hematocrit samples were removed at the indicated time points and evaluated by Giemsa-stained thin smear microscopy (*top*) and RT-PCR (*bottom*) for the three 18S rRNA, PF3D7_0630000 and pfs25 targets. Duplicate samples were tested for 18S rRNA (*black squares*) and PF3D7_0630000 (*red diamonds*) with 95% CIs indicated; singlet samples were tested for positive control gametocyte marker pfs25 (*grey triangles*). Fold change was calculated for each target as the ratio of the expression on the day of interest relatives to expression of the same target on Day 6 of gametocyte culture

To study the time course of PF3D7_0630000 expression, samples were taken from gametocyte cultures during Days 6–14 of culture and compared to expression of the Day 6 sample. This approach was used because on the sixth day of culture, a mix of residual asexual stages and immature gametocytes present, but after the tenth day of culture, most of the parasites were mature gametocytes with ring, trophozoite and schizont stages no longer observed. Unlike asexual-stage parasites that do not express the PF3D7_0630000 spliced mRNA, gametocytes express both pfs25 and PF3D7_630000 mRNAs from by the sixth day of culture. Expression of both targets increased as the cultures matured with pfs25 expression exceeding that of PF3D7_0630000 (Fig. 5). At peak expression (day 14), pfs25 was increased by >2000-fold compared to day 6 of culture while PF3D7_0630000 increased >25-fold.

### Detection of gametocytaemia in a volunteer in a controlled human malaria infection trial

**Fig. 6:**
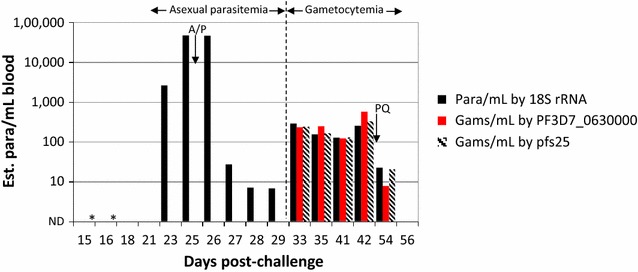
Course of molecular target detection in infected subject. A patient infected with *P. falciparum* by CHMI developed late-onset, symptomatic, blood-smear positive (D25) peripheral infection and was treated 25 days post-challenge with a standard treatment course of atovaquone–proguanil (A/P). Following treatment, symptoms resolved but the patient continued to display 18S rRNA positivity. pfs25 and PF3D7_0630000 RT-PCRs were performed and demonstrated a period of gametocyte (Gams)-specific mRNA positivity during the secondary wave of 18S rRNA positivity, consistent with gametocytaemia. The 18S rRNA-based results were unaffected by DNase treatment (not shown). Stage-specific 18S rRNA conversion factors were used to estimate parasite densities (7.4 × 10^3^ copies of 18S rRNA/asexual ring *left* of *vertical line*; 4.5 × 10^4^ copies of 18S rRNA/mature gametocyte *right* of *vertical line*) and standard curve of pure cultured gametocytes diluted into whole blood was used for quantification of gametocyte-specific qRT-PCRs. Primaquine (PQ, 45 mg oral) was administered one time 45 days post-challenge. *ND,* not detected; *asterisk* pfs25 RT-PCR not performed

To determine if the novel spliced gametocyte-specific mRNA RT-PCR could detect low level gametocytaemia from clinical specimens, samples were first evaluated from a clinical trial in a Seattle-based controlled human malaria infection trial evaluating the prophylactic activity of the drug DSM265. This placebo-controlled prophylactic drug trial was blinded at the time of this manuscript, and it was thus unknown whether subjects received the active compound or the placebo control. Subjects were challenged with 3200 PfSPZ (Sanaria, Inc., Rockville, MD) on Day 0. Final details of the ongoing DSM265 study will be published in a separate manuscript at a later date and do not affect the data described below. *Plasmodium* infection status was monitored by daily 18S rRNA RT-PCR. One subject developed a late onset infection with 18S rRNA qRT-PCR positivity starting on Day 23 (Fig. 6). The subject was symptomatic (grade 1–2 nausea, vomiting, headache) during this time and was thick blood smear positive on Day 25. The subject was treated on Day 25 with atovaquone–proguanil with rapid conversion to negative blood smears and resolution of symptoms. *Plasmodium* 18S rRNA concentrations dropped following treatment but did not reach undetectable levels. On Day 33 post-challenge, resurgence in *Plasmodium* 18S rRNA was noted although the subject was asymptomatic. Persistent asymptomatic positivity for *Plasmodium* 18S rRNA continued from days 33–42 post-challenge. Neither pfs25 nor PF3D7_0630000 mRNAs were detected during the symptomatic period but both were detected during the second wave of asymptomatic parasitaemia (Fig. 6). The patient was subsequently treated orally with 45 mg primaquine and was negative for all biomarkers at 56 days post-challenge.

### Multiplex comparison against other reported gametocyte-specific spliced mRNA targets

In addition to PF3D7_0630000, three other spliced mRNA targets have been reported: the *P. falciparum* meiotic recombination protein DMC1-like protein gene (PF3D7_0816800) [26], an immature gametocyte marker PHISTa of unknown function (PF3D7_1477700/PF14_0748) [27] and a mature gametocyte marker of unknown function (PF3D7_1438800/PF14_0367) [27]. Each of these targets were individually multiplexed with the pan-*Plasmodium* 18S rRNA assay and tested against whole blood samples spiked with high (4 × 10^5^ parasites/mL) or low (8 × 10^2^) density pure cultured ring-stage samples or with day 14 cultured gametocytes (Table 3). The PF3D7_1477700 and PF3D7_1438800 markers [27] could not be successfully multiplexed with the pan-*Plasmodium* 18S rRNA RT-PCR because false positive results were obtained for asexual samples at annealing temperatures of 50 °C (standard for 18S rRNA RT-PCR) and 60 °C (as originally reported for these gametocyte markers [27]). False positive results for the PF3D7_1477700 and PF3D7_1438800 markers were also obtained when testing singleplex reactions against asexual stage parasites (data not shown). PF3D7_0630000 and pfs25, the spliced PF3D7_0816800 target were positive only in the presence of gametocytes (Table 3).

**Table 3:** Example cycle thresholds for PF3D7_0630000 and published gametocyte-specific RT-PCRs

RT-PCR target (annealing temp.)	Asexual		Gametocyte 3 × 10^6^ g/mL	Negative control
	4 × 10^5^ p/mL	8 × 10^2^ p/mL		
PF3D7_0630000 (50 °C)	ND	ND	23.02	ND
PF3D7_0816800 (50 °C)	ND	ND	24.98	ND
PF3D7_1477700 (50 °C)	31.96	ND	25.99	ND
PF3D7_1477700 (60 °C)	31.67	38.74	26.50	ND
PF3D7_1438800 (50 °C)	35.42	ND	24.94	ND
PF3D7_1438800 (60 °C)	33.48	ND	25.51	ND
pfs25 (50 °C, DNased template)	ND	ND	17.67	ND
*Plasmodium* 18S rRNA	14.91	23.82	10.20	ND

*ND* not detected

### One-step multiplex detection of gametocytes in asymptomatic subjects in Uganda

**Fig. 7:**
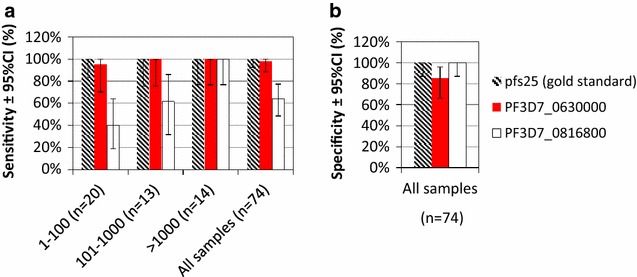
Detection of gametocyte-specific markers in Ugandan field samples. **a** Sensitivity analysis of candidate multiplexed 18S rRNA and gametocyte-specific RT-PCRs as compared to a multiplex assay for 18S rRNA and pfs25 mRNA. Results based on 74 samples from asymptomatic subjects. **b** Specificity analysis of the same samples. Data is in Additional file 3

Multiplex qRT-PCRs combining *Plasmodium* 18S rRNA with either PF3D7_0816800, PF3D7_0630000 or pfs25 were evaluated against 74 *Plasmodium*-infected samples collected from asymptomatic human volunteers in a Ugandan field study. The parasite densities of these samples ranged from 25 to 2.8 × 10^7^ parasites/mL with estimated gametocyte densities ranging from 1 to 156,828 gametocytes/mL (based on pfs25 standard curves). The pfs25 multiplex assay detected gametocytes in 47/74 samples, PF3D7_0630000 in 50/74 samples and PF3D7_0816800 in 30/74 samples (Additional file 3). pfs25 and PF3D7_0630000 showed a high degree of agreement. Compared to the pfs25 gold standard, the sensitivity of PF3D7_0630000 for gametocyte detection was 97.9% (95% CI 88.7–100.0%) and specificity 85.2% (95% CI 66.3–95.8%). The sensitivity of PF3D7_0816800 qRT-PCR was 63.8% (95% CI 48.5–77.3%) and specificity 100% (87.2–100.0%). Whereas the PF3D7_0630000 reagents detected gametocytes across all pfs25-positive gametocyte densities, PF3D7_0816800 RT-PCR was less sensitive at densities <1000 gametocytes/mL but was positive in all samples with a density of >1000 gametocytes/mL (Table 4; Fig. 7).

**Table 4:** Performance of gametocyte-specific RT-PCR markers against field samples from Uganda

Gametocytes/mL	n	pfs25	PF3D7_0630000	PF3D7_0816800
Sensitivity (95% CI)				
1–100 (n = 20)	20	100% (83.2–100.0%)	95.0% (75.1–99.9%)	40.0% (19.1–64.0%)
101–1000 (n = 13)	13	100% (75.3–100.0%)	100.0% (75.3–100.0%)	61.5% (31.6–86.1%)
>1000 (n = 14)	14	100% (76.8–100.0%)	100.0% (76.8–100.0%)	100.0% (76.8–100.0%)
All samples (n = 74)	74	100% (92.5–100.0%)	97.9% (88.7–100.0%)	63.8% (48.5–77.3%)
Specificity (95% CI)				
All samples (=74)	74	100% (87.2–100.0%)	85.2% (66.3–95.8%)	100.0% (87.2–100.0%)

Sensitivity = true positive/(true positive + false negative)

Specificity = true negative/(true negative + false positive)

Gametocytes/mL quantities were based on pfs25 standard curve

Quantification of gametocytes in field samples based on pfs25 resulted in estimated densities 1.18 log_10_ parasites/mL (95% CI 0.89–1.46 log_10_ parasites/mL) lower on average than the paired estimates made with PF3D7_0630000. Lower pfs25-derived gametocyte quantification could not be explained by overall loss of total nucleic acids during DNase treatment since 35/39 evaluable paired samples (positive by pfs25 and PF3D7_0630000) actually showed earlier 18S rRNA C_T_s in the pfs25 sample compared to the PF3D7_0630000 sample (Additional file 3).

## Discussion

*Plasmodium* gametocytes can be detected with mRNA-based molecular methods at sub-microscopic densities. Commonly targeted gametocyte-specific *P. falciparum* mRNAs include those made from single exon genes *pfs25* and *pfs230* [17–20], which necessitates DNase treatment prior to RT-PCR. Here, based on published RNAseq data [28], 18 gametocyte-specific, multi-exon mRNAs were identified and evaluated as DNase-free gametocyte targets to determine if they could be multiplexed with an existing highly sensitive *Plasmodium* 18S rRNA qRT-PCR. The bioinformatics strategy employed here selected for genes that were highly expressed in mature gametocytes and showed near-zero sense or antisense expression in asexual stages. In wet-lab testing, two female gametocyte-specific mRNAs showed suitable multiplex RT-PCR target characteristics: PF3D7_0630000 and PF3D7_0514500. PF3D7_0630000 encodes a CPW-WPC protein likely expressed in ookinetes, though the coding mRNAs are first expressed and post-transcriptionally regulated in gametocytes [38]. Since the PF3D7_0630000 mRNA accumulates in mature female gametocytes as the parasite awaits ookinete formation, this target may be an ideal gametocyte marker in human blood. PF3D7_0514500 (alias PFE0725C) encodes a six-exon conserved membrane protein of unknown function and was noted to be a member of the sexual development gene cluster in a previous full-genome high-density oligonucleotide microarray study [39]. Interestingly, PF3D7_0630000 was absent from the same gene cluster study.

Since PF3D7_0630000 was the most promising target, it was intended to be compared against RT-PCR assays for pfs25 and for three other known spliced gametocyte-expressed mRNAs: PF3D7_0816800, PF3D7_1477700 and PF3D7_1438800. However, two of the intended comparator targets could not be multiplexed with the 18S rRNA RT-PCR assay and were therefore not studied against PF3D7_0630000. These targets produced positive results when tested against microscopically-pure asexual stage cultures (that were also pfs25-negative). The extraction method and RT-PCR mastermix used here differed from that originally reported for these markers [27], which may account for discrepancies. In addition, unlike the other targets studied in this project, the PF3D7_1477700 and PF3D7_1438800 mRNAs were both male gametocyte-specific [37], which could have led to false positives in asexual samples if male gametocytes (or less apparent exflagellated forms) were present at minuscule concentrations in asexual cultures; such forms would also be pfs25-negative. While PF3D7_1477700 and PF3D7_1438800 mRNAs may be suitable for multiplexing with 18S rRNA in another extraction/mastermix system, this possibility was not further evaluated here.

The PF3D7_0630000 multiplex RT-PCR was comparably sensitive to pfs25 when tested against a samples from asymptomatic Ugandan subjects. Given this performance, PF3D7_0630000 RT-PCR will be able to detect gametocytes at densities that contribute to transmission, although this should be studied in future in prospective studies. Interestingly, PF3D7_0630000-derived gametocyte density estimates were 1.18 log_10_ higher on average than pfs25-based estimates, which likely reflects degradation of pfs25 mRNA (but not more robust 18S rRNA) during the DNase treatment step. Detection of pfs25 mRNA by QT-NASBA (which does not require DNase treatment) was reportedly more sensitive than pfs25 RT-PCR, and the authors postulated that the DNase treatment step could have reduced the sensitivity of pfs25 RT-PCR [12]. Thus, it is likely that the increased sensitivity of pfs25 RT-PCR (due to its increased expression) is somewhat offset by increased degradation at the DNase treatment step.

By eliminating DNase treatment, gametocyte-specific RT-PCR targets can be directly incorporated into multiplex RT-PCR assays from total nucleic acids. This importance of this workflow improvement may be significant. Although ‘on-column’ DNase treatment is available for some manual RNA purification kits, this was not an option for the platform used here (Abbott m2000sp/rt [22]. Elimination of DNase treatment would be advantageous. In epidemiological studies, simplified sample processing that minimizes hands-on time and eliminates manual steps are desirable since extra steps serve to increase false positive and negative results through cross-contamination, target degradation and other processing errors. The use of spliced gametocyte-specific mRNAs such as those identified here (PF3D7_0630000 and PF3D7_0514500) or those reported previously [26, 27] offer the possibility of this sort of simplified testing. Other spliced mRNA targets beyond these may also be suitable for RT-PCR. Similarly, spliced mRNA RT-PCR may also be useful for detecting asexual stage-specific spliced mRNAs such as the ring-specific transcript from the two-exon PF3D7_0501300 (PFE0065w) gene previously used as an asexual parasite marker [27]. Multiplex assays that include the *Plasmodium* 18S rRNA, a spliced gametocyte-specific mRNA and a spliced ring/asexual-specific mRNA could eventually provide for one-step *P. falciparum* infection monitoring that would differentiate between potentially symptomatic and asymptomatic infections. The PF3D7_0630000-specific spliced marker identified here may be useful in future gametocyte screening studies.

## Additional files

**Additional file 1.** Expression data on selected genes (from plasmodb based on genes in [28]). Contains expression data for selected stage-specific mRNA transcripts.

**Additional file 2.** Candidate primer pairs evaluated by LCGreen RT-PCR. Contains forward and reverse primer sequences for candidate targets and ΔC_T_ values for stage-specificity.

**Additional file 3.** Data and 2X2 files for field study samples. Contains the raw data from multiplex RT-PCR assays performed on the samples from asymptomatic subjects in a field study in Uganda and 2X2 sensitivity/specificity tables.
